# Influence of genetic diversity of seventeen *Beauveria bassiana* isolates from different hosts on virulence by comparative genomics

**DOI:** 10.1186/s12864-020-06791-9

**Published:** 2020-06-30

**Authors:** Zhengkun Zhang, Yang Lu, Wenjing Xu, Li Sui, Qian Du, Yangzhou Wang, Yu Zhao, Qiyun Li

**Affiliations:** Jilin Key Laboratory of Agricultural Microbiology, Key Laboratory of Integrated Pest Management on Crops in Northeast China, Ministry of Agriculture, Changchun, 130033 Jilin Province P. R. China

**Keywords:** Whole genome re-sequencing, *Beauveria bassiana*, Genetic diversity, Virulence

## Abstract

**Background:**

*Beauveria bassiana* (*B. bassiana*) is a famous entomopathogenic fungus that could parasitize on hundreds of insect species, which are being used as an environmentally friendly mycoinsecticide. Nevertheless, the possible effect of genetic diversity of these *B. bassiana* isolates from different hosts on virulence has not been explored before. In order to explore that issue, we compared the genome sequences among seventeen *B. bassiana* isolates from 17 different insects using whole genome re-sequencing, with *B. bassiana* strain ARSEF 2860 as the reference genome.

**Results:**

There were a total of 10,098 missense mutated genes, 720 positively selected genes were identified in 17 strains of *B. bassiana*. Among these, two genes with high frequency mutations encode the toxin-producing non-ribosomal peptide synthase (NRPS) protein. Seven genes undergoing positive selection were enriched in the two-component signaling pathway that is known to regulate the fungal toxicity. In addition, the domain changes of three positively selected genes are also directly related to the virulence plasticity. Besides, the functional categorization of mutated genes showed that most of them involved in the biological functions of toxic proteins involved in.

**Conclusions:**

Based on our data, our results indicate that several mutated genes and positively selected genes may underpin virulence of *B. bassiana* towards hosts during infection process, which provide an insight into the potential effects of natural variation on the virulence of *B. bassiana*, which will be useful in screening out potential virulence factors in *B. bassiana*.

## Background

*Beauveria bassiana* (*B. bassiana*), a well-known entomopathogenic fungus, is belonged to the family Clavicipitaceae, order Hypocreales [1]. Owing to the high genetic variability, *B. bassiana* exhibits high adaptability to various environmental conditions and thus could parasitize a wide range of insect populations [2]. Currently, *B. bassiana* is actively developed as a biological control tool for various insects in all regions of world [3, 4]. According to Li et al. report, approximately one million hectares are usually treated with *B. bassiana* to reduce the occurrence of forest insects in China every year [5]. Generally, *B. bassiana* is thus a generalist with no strict host preference and can be used as an effective and sustainable biological control agent against a myriad of insect pests [6].

Virulence is variable and not universal for a prescribed host and pathogen [7]. Increasing studies have confirmed that the virulence of *B. bassiana* strains from a specific insect host varies frequently [8]. It has been recognized that the strain of *B. bassiana* isolated from a given insect showed low virulence against other insects, and showed stronger virulence against these insects after domestication [9], indicating that the virulence of *B. bassiana* to hosts has a great plasticity. Genome structure and DNA sequence variation in these isolates could be related to many factors in the biology of this fungus. Recently, the re-sequencing of numerous individuals within pathogen species has been as a forceful tool to explore the genetic mechanism in fungal and fungal-host interaction studies [10]. For example, Mburu et al. compared the gene sequences of two *B. bassiana* isolates with different levels of virulence and repellence to the termite, and then some differences were found in the nucleotide sequences of the two isolates of *B. bassiana*, suggesting a genetic basis for the observed intra-specific differences in their virulence against the termite [11]. Valero-Jiménez et al. sequenced the genomes of five isolates of *B. bassiana* with low/high virulence, and finally identified several genes and molecular processes that effect the virulence towards Mosquitoes [12]. These studies provide a better understanding of the natural variation in virulence by genome analysis. Thus, understanding genetic polymorphisms is helpful to understand its potential virulence diversity of *B. bassiana* isolates isolated from different hosts.

In the present study, we aim to gain our knowledge in understanding genomic diversity involved in virulence and host-pathogen interaction for 17 *B. bassiana* strains by a comparative genomics analysis. Thus, we sequenced the 17 *B. bassiana* isolates from different hosts compared to the reference genome *B. bassiana* strain ARSEF 2860. Based on the analysis, understanding the potential factors of genetic variation on the virulence of *B. bassiana* will improve our methods to use this fungus as an effective and sustainable biological control agent against.

## Results

### Sequencing and mapping summary

Quality control results of sequencing data of 17 *B. bassiana* strains were summarized in Table S1. Finally, a total of 160 Gb containing 459 million raw reads were produced, with an average of 27,048,202 reads for each sample. After filtering low quality reads and duplicate reads, 396 million high quality reads were extracted. Approximately 71.22% of the cleaned reads could be successfully mapped to the *B. bassiana* strain ARSEF 2860 reference genome, with varying 67.30 to 75.30% among different strains (Table 1). These mapped sequences were used for subsequent analyses.

**Table 1:** Comparison results of sequenced reads of 17 *B. bassiana* isolates aligned to the reference genome of *B. bassiana* strain ARSEF 2860

Sample	Clean reads	Mapped-reads	Left mapped reads	Right mapped reads	Pair mapped reads	Map rate
S2	5,787,346	3,894,494	1,160,251	2,734,243	1,179,149	0.672932636
S3	4,753,518	3,307,827	1,063,104	2,244,723	1,078,463	0.695869249
S4	6,475,936	4,471,055	1,421,647	3,049,408	1,443,532	0.690410622
S5	8,196,298	5,515,918	1,632,128	3,883,790	1,662,691	0.672976751
S6	8,562,752	5,769,931	1,720,400	4,049,531	1,749,434	0.673840723
S7	12,633,678	9,050,879	3,206,347	5,844,532	3,251,762	0.716408872
S8	19,833,546	13,991,144	4,814,073	9,177,071	4,879,648	0.705428268
S9	25,549,484	18,221,368	6,473,684	11,747,684	6,551,046	0.713179491
S10	15,359,600	11,307,010	4,283,183	7,023,827	4,324,718	0.736152634
S11	17,281,180	12,077,193	4,060,203	8,016,990	4,121,883	0.698863909
S13	15,289,798	11,493,464	4,526,481	6,966,983	4,559,965	0.751708034
S14	20,971,244	15,334,454	5,743,802	9,590,652	5,793,930	0.73121337
S15	34,505,488	25,035,584	9,278,608	15,756,976	9,385,046	0.725553686
S18	30,045,320	22,624,755	9,075,195	13,549,560	9,134,172	0.753020936
S19	63,064,590	45,538,739	17,114,002	28,424,737	17,242,056	0.722096806
S20	60,849,634	44,257,288	16,899,228	27,358,060	17,070,101	0.727322173
S21	44,537,158	32,079,845	11,709,427	20,370,418	11,832,010	0.72029394

### SNPs and InDels identification

**Fig. 1:**
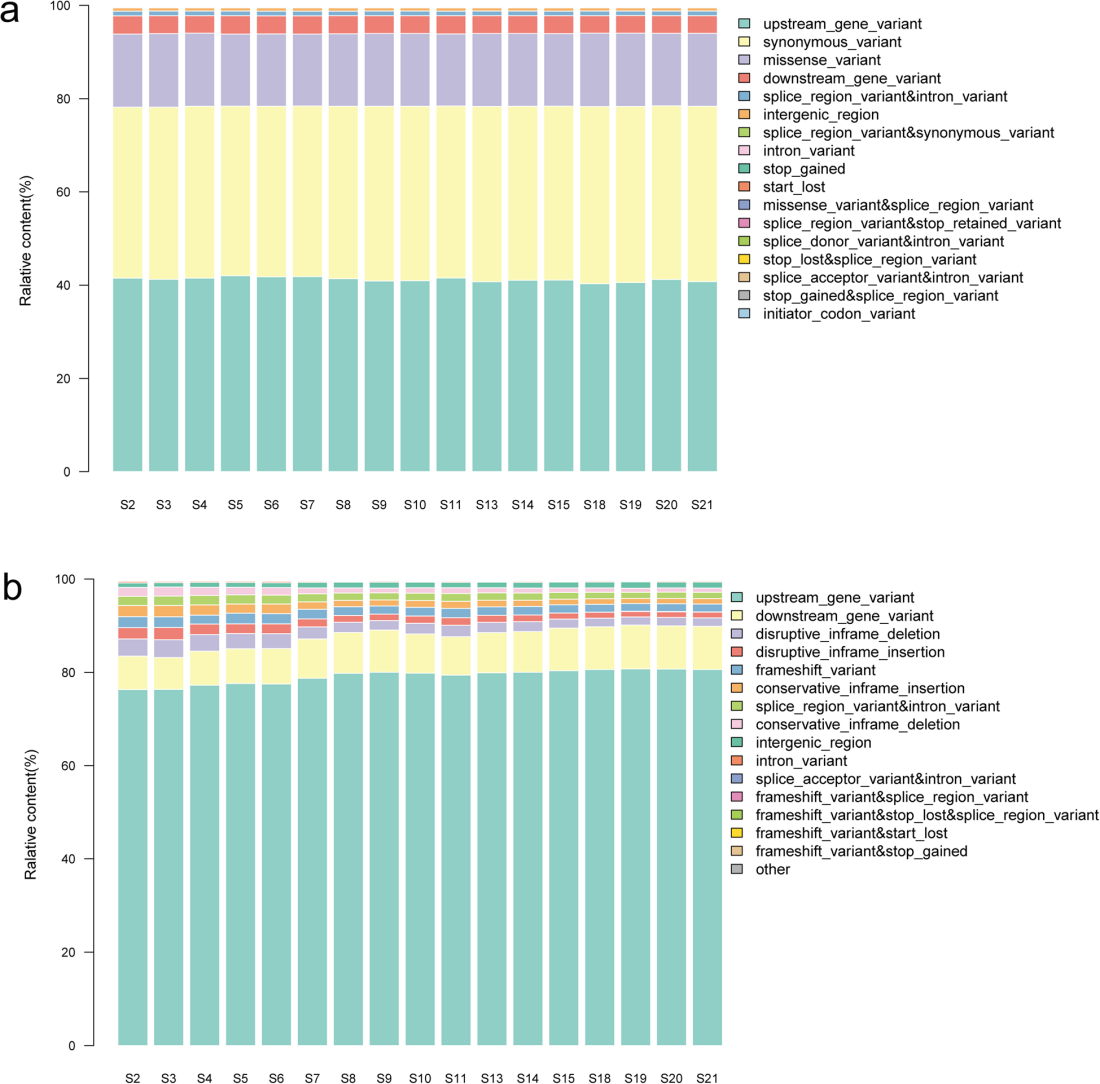
The stacked-column displays the distribution of SNP-types (**a**) and Indel-types (**b**) in each *B. bassiana* isolate genotype. The horizontal axis represents 17 samples of *B. bassiana*, and the vertical axis represents the relative content of different SNP/Indel distribution types. The number of genes shared between the 17 isolates presented in each category in Table S5

All clean reads were aligned to the reference genome assembly of *B. bassiana* strain ARSEF 2860. As a result, a total of 2,779,949 SNPs were identified across 17 *B. bassiana* isolates genotypes, among which 1,959,716 (70.49%) are synonymous and 820,233(29.51%) are nonsynonymous. In addition, a total of 884,811 Indels were obtained, with 423,409 (47.85%) InDel-Ins and 461,402 (52.15%) InDel-Del (Table 2). The distributions of SNP-types and Indel-types of 17 samples were obtained, it showed that majority of SNP variation were belonged to different types including the upstream gene variants (40.82%), the synonymous variants (37.54%), and the missense variants (15.55%) (Fig. 1a). Most of the InDels variation were located in the upstream region of the genes (79.15%) (Fig. 1b). At last, we detected 7421 missense genes are shared by all strains, accounting for 73.49% of all missense genes (10,098).

**Table 2:** Number of mutations observed in the seventeen *B. bassiana* isolates comparing with the reference genome of *B. bassiana* strain ARSEF 2860

Sample	No. synonymous SNPs	No. non-synonymous SNPs	Syn/Nonsyn	SV	InDel-Ins	InDel-Del
S2	115,930	49,487	2.342635	48	10,119	11,527
S3	114,965	48,966	2.347854	57	9,573	10,697
S4	117,291	49,700	2.35998	27	12,343	14,010
S5	117,039	49,616	2.358896	120	13,138	14,883
S6	116,739	49,348	2.365628	144	14,464	16,244
S7	117,098	49,273	2.376515	132	21,341	23,608
S8	117,232	49,111	2.387082	333	27,016	29,492
S9	115,755	48,153	2.4039	247	30,211	32,644
S10	116,014	48,168	2.408528	185	26,230	28,652
S11	117,508	49,104	2.393043	179	23,756	26,421
S13	115,278	47,742	2.414603	201	27,836	30,314
S14	116,620	48,574	2.400873	323	29,081	31,729
S15	115,628	47,972	2.410323	333	33,174	35,704
S18	114,229	47,079	2.426326	363	34,894	37,471
S19	107,452	44,572	2.410751	563	37,946	40,478
S20	111,089	46,145	2.40739	400	36,400	39,130
S21	113,849	47,223	2.41088	508	35,887	38,398

### Functional annotation

**Fig. 2:**
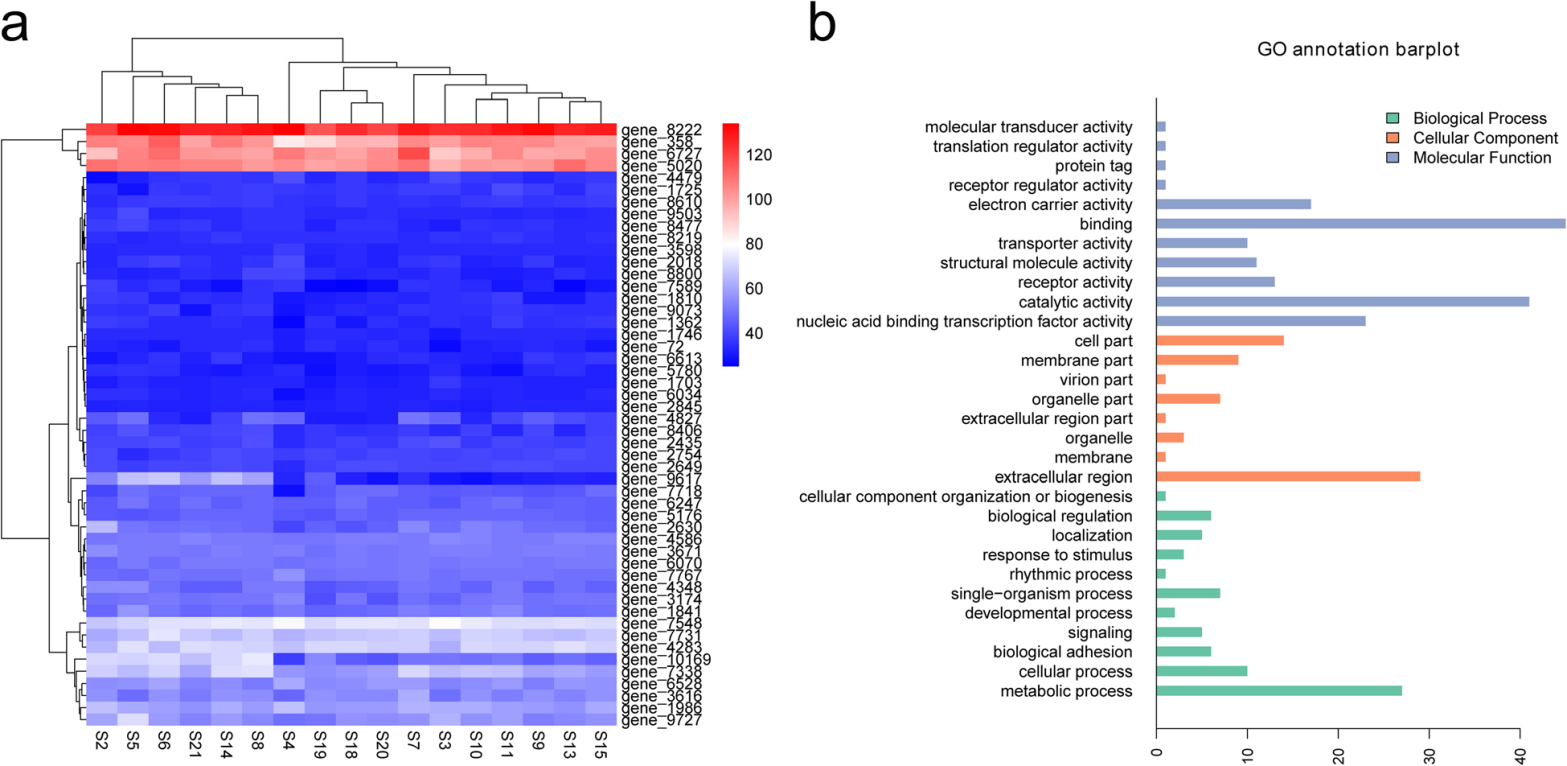
The mutation numbers and annotation information of 50 genes with high frequency of missense mutations. **a** A hierarchical clustering analysis showing the relative difference in the number of SNPs in these top 50 genes in 17 *B. bassiana* samples. The red 4 genes exhibit a higher number of SNP mutations in 17 *B. bassiana* samples, while the blue 46 genes exhibit a low number of SNP mutations in 17 *B. bassiana* samples. **b** Gene Ontology (GO) functional annotation of the top 50 genes with missense mutations in the re-sequencing data of 17 *B. bassiana* isolates. The results are summarized in three main categories: biological process (BP), cellular component (CC) and molecular function (MF)

A hierarchical clustering analysis of the top 50 high frequency missense mutation genes in 17 *B. bassiana* samples was conducted (Fig. 2a). Clearly, there was no significant numerical difference of each gene in different samples sequenced in this study. However, among different genes, more variation was relatively abundant in four genes (gene_8222, gene_358, gene_6727, and gene_5020) but few variations were relatively lacking in other 46 genes in all *B. bassiana* isolates. Next, we determined the functional categories of these top 50 genes using Gene Ontology database, then their functions were belonged to three general directions. In the biological process category, the top three highly enriched GO terms included binding, catalytic activity, and nucleic acid binding transcription transporter activity. The GO terms of extracellular region, cell part, membrane part, and organelle part were significantly enriched in the cellular component. The molecular function category showed a high percentage of metabolic process, cellular process, and single-organism process (Fig. 2b).

### Species clustering analysis

**Fig. 3:**
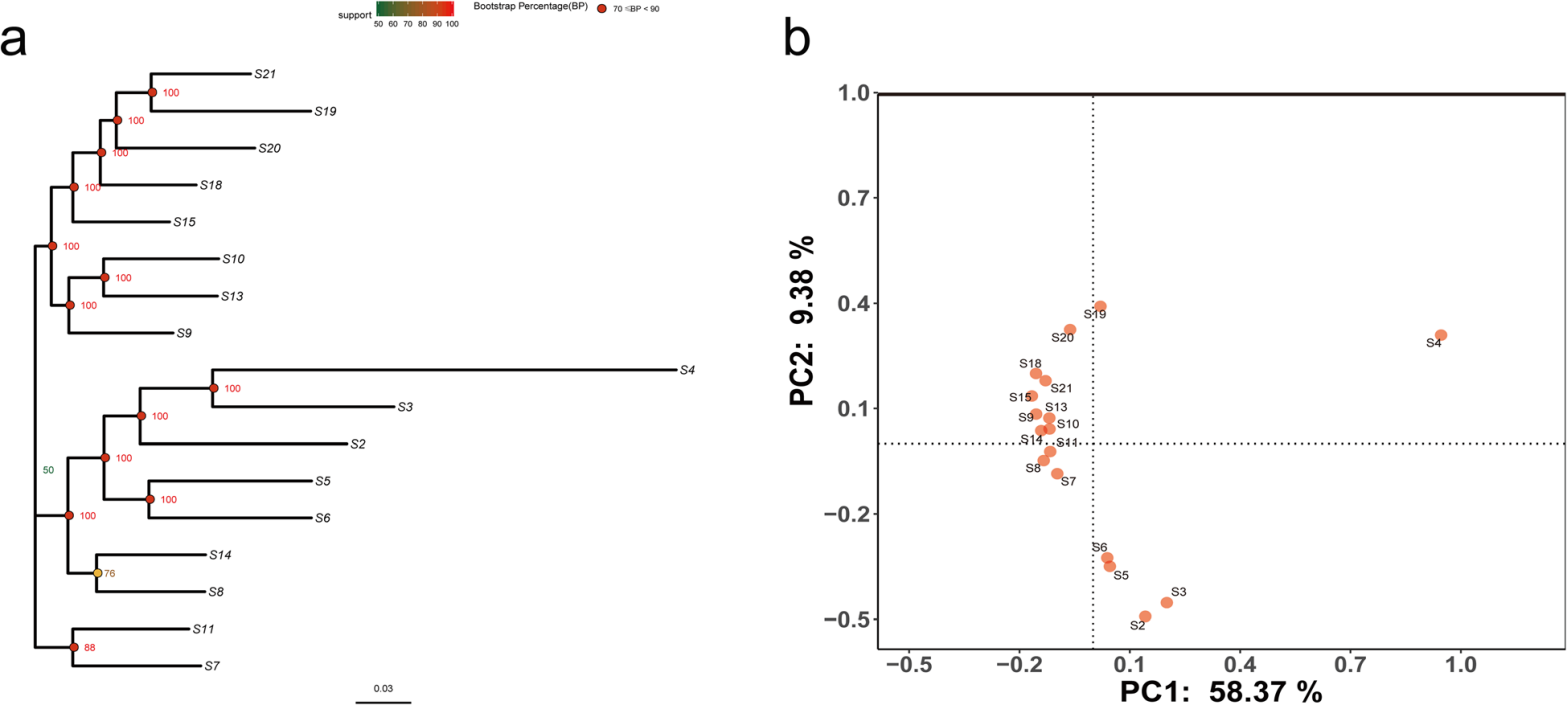
The genetic diversity of the missense SNPs in the re-sequencing data of seventeen *B. bassiana* isolates. **a** A phylogenetic tree was constructed. All missense mutated nucleotide sequences of 17 strains of *B. bassiana* were used to construct an evolutionary tree based on the similarity of SNP missense mutations according to modelfinder and iqtree software. The phylogenetic tre*e* was constructed with 100 bootstrapping replicates. Similar missense mutations were distributed in the same cluster. **b** Principal component analysis (PCA) analysis of 17 samples based on missense SNPs. Principle component plot constructed by Plink software

To observe the divergence contained in the individual SNPs among the 17 *B. bassiana* isolates at the genomic level, we constructed a phylogenetic tree based on the full coding sequence of the genes containing the SNPs obtained from the sequence comparisons. As shown in Fig. 3a, three separate clusters were generated including the isolates of S2\S3\S4\S5\S6\S8\S14, the isolates of S9\S10\S13\S15\S18\S19\S20\S21, and the isolates of S7/S11. Besides, we performed a principal component analysis (PCA) to examine the genetic relationships among these 17 samples, a clearer separation of three clusters was constructed (Fig. 3b).

### Ka/Ks analysis

**Fig. 4:**
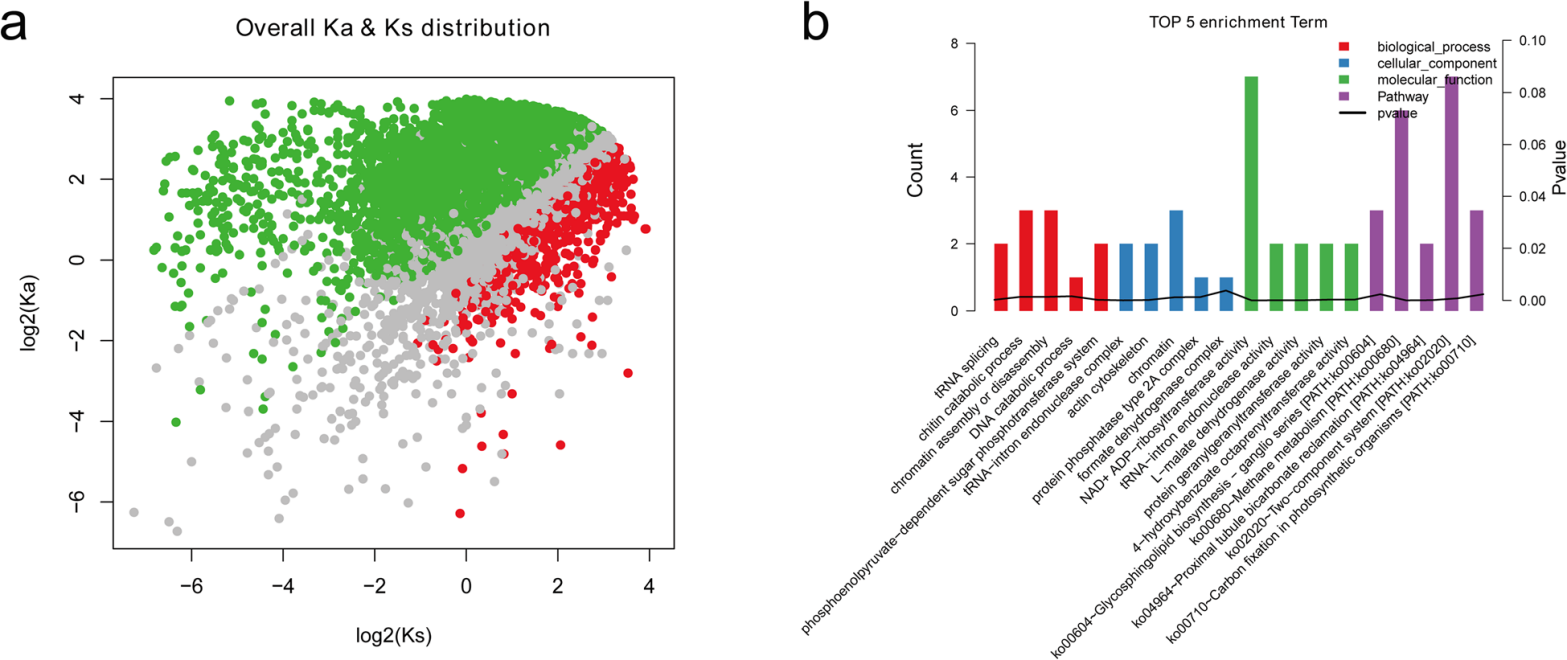
Ka (non-synonymous)/Ks (synonymous) analysis and functional annotation of the genes with missense mutations in the re-sequencing data of 17 *B. bassiana* isolates. **a** The overall Ka/Ks distribution. The red dots represent genes with positive selection in 17 samples, while the green dots represent genes with negative selection in 17 samples. **b** Gene Ontology (GO) and Kyoto Encyclopedia of Genes and Genomes (KEGG) functional enrichment analysis of 720 positive selection genes. The GO annotation results were summarized in three main categories: biological process (BP), cellular component (CC) and molecular function (MF). The KEGG annotation results were summarized in pathways

To confirm the genes subjected to significant selection in of *B. bassiana* domestication, we estimated the statistic Ka/Ks with the 10,098 genes contained missense mutation in re-sequencing data of 17 *B. bassiana* isolates. Finally, a total of 7050 and 720 genes are respectively showed with negative selection or positive section (strong purifying selection) in seventeen samples (Fig. 4a). Subsequently, GO and KEGG pathway analyses were conducted on the positively selected genes. GO enrichment analysis showed highlight a significant enrichment in genes involved in NAD + ADP-ribosyltransferase activity, chromatin, chitin catabolic process, chromatin assembly or disassembly, phosphoenolpyruvate-dependent sugar phosphotransferase system, tRNA-intron endonuclease complex, actin cytoskeleton, tRNA-introendonuclease activity, L-malate dehydrogenase activity, protein geranygeranyltransferase activity, 4-hydroxybenzoate octaprenyltransferase activity, and so on (Fig. 4b). The KEGG pathway analysis revealed that the genes were overrepresented in 5 pathways, of which the highest represented pathway is glycosphingolipid biosynthesis-ganglio series (PATH: ko00604), followed by methane metabolism (PATH: KO00680), proximal tubule bicarbonate reclamation (PATH: KO04964), two component systems (TCSs) (PATH: KO02020), and carbon fixation in photosynthetic organisms (PATH: ko00710) (Fig. 4b). Herein, a total of seven genes (gene_664, gene_2709, gene_3056, gene_3362, gene_7254, gene_7433, gene_8336) with positive selection were abundantly enriched in this pathway, and the proteins encoded by these genes were Oxidoreductase short-chain dehydrogenase/reductase family, Galactoside O-acetyltransferase, Ulp1 protease family protein, UcrQ family protein, Acyl-CoA N-acyltransferase, Ubiquinol-cytochrome c reductase complex subunit, and CAAX amino terminal protease (Table 3). The corresponding mutation information of these seven genes was presented in Table S2.

**Table 3:** The information of the seven positively selected genes that are enriched in the two component systems

Gene	Protein-description	Protein	EC	KO
gene_664	Oxidoreductase short-chain dehydrogenase/reductase family	XP_008593983.1	1.1.1.-	K18347
gene_2709	Galactoside O-acetyltransferase	XP_008596028.1	2.3.1.-	K15853
gene_3056	Ulp1 protease family protein	XP_008596375.1	3.4.22.-	K12292
gene_3362	UcrQ family protein	XP_008596681.1	1.10.2.2	K00411
gene_7254	Acyl-CoA N-acyltransferase	XP_008600573.1	2.3.1.-	K15853
gene_7433	Ubiquinol-cytochrome c reductase complex subunit	XP_008600752.1	1.10.2.2	K00411
gene_8336	CAAX amino terminal protease	XP_008601655.1	3.4.22.-	K12292

### Structural variations detection

The detection of SVs was achieved by making use of BreakDancer programs. A total of 4163 individual SVs were predicted, with an average of 245 SVs in each *B. bassiana* strain genotype (Table 2). Most of the SVs were unique in each sample, and only 6 structural variation loci were shared among different samples, as shown in Table S3.

### Protein domain analysis

Finally, we investigated the effect of the missense mutations of the 720 positively selected genes on protein structure by the comparation of 17 samples. Collectively, 13 putative domains encoded by 13 protein-coding genes were identified, of which the genome regions encoding proteins with MutS_IV domain, Zn_clus domain, Fungal_trans_2 family, Metallophos domain, and Metallophos_2 domain were most richest and shared by seventeen samples (Table 4).

**Table 4:** Genes and domain prediction information for domain changes in the seventeen *B. bassiana* isolates

Gene ID	Hmm_ accession	Hmm_name	Type	Compare with reference	Samples
gene_10,314	PF05190.15	MutS_IV	Domain	lost	S10; S11; S13; S14; S15; S18; S19; S2; S20; S21; S3; S4; S5; S6; S7; S8; S9
gene_3355	PF13637.3	Ank_4	Domain	lost	S4
gene_3355	PF13857.3	Ank_5	Domain	add	S4
gene_3930	PF00172.15	Zn_clus	Domain	add	S10; S11; S13; S14; S15; S18; S19; S2; S20; S21; S5; S6; S7; S8; S9
gene_3930	PF00172.15	Zn_clus	Domain	add	S3
gene_3930	PF00172.15	Zn_clus	Domain	add	S4
gene_3930	PF11951.5	Fungal_trans_2	Family	add	S10; S11; S13; S14; S15; S18; S19; S2; S20; S21; S3; S5; S6; S7; S8; S9
gene_3930	PF11951.5	Fungal_trans_2	Family	add	S4
gene_3959	PF00023.27	Ank	Repeat	lost	S11; S5; S6
gene_4478	PF00023.27	Ank	Repeat	lost	S11; S15; S19; S4; S9
gene_4478	PF12796.4	Ank_2	Repeat	lost	S10; S20; S21; S3; S6
gene_5131	PF00023.27	Ank	Repeat	lost	S14; S15; S18; S8; S9
gene_5131	PF13857.3	Ank_5	Domain	add	S10; S11; S14; S15; S2; S3; S5; S8; S9
gene_5131	PF13857.3	Ank_5	Domain	add	S18; S20
gene_5369	PF00149.25	Metallophos	Domain	lost	S10; S11; S13; S14; S15; S18; S19; S2; S20; S21; S3; S4; S5; S6; S7; S8; S9
gene_5369	PF12850.4	Metallophos_2	Domain	add	S10; S11; S13; S14; S15; S18; S19; S2; S20; S21; S4; S5; S7; S8; S9
gene_5369	PF12850.4	Metallophos_2	Domain	add	S3; S6
gene_5651	PF01636.20	APH	Family	add	S13; S18
gene_7731	PF00023.27	Ank	Repeat	add	S4
gene_9165	PF13414.3	TPR_11	Repeat	add	S10; S11; S13; S14; S15; S18; S19; S2; S20; S21; S4; S6; S7; S8; S9
gene_9165	PF13414.3	TPR_11	Repeat	add	S3
gene_9207	PF13176.3	TPR_7	Repeat	add	S18
gene_9900	PF00023.27	Ank	Repeat	lost	S10; S18; S20; S21; S4; S9
gene_9932	PF13516.3	LRR_6	Repeat	add	S11; S21; S3

## Discussion

*B. bassiana*, originated from the different hosts shows distinct virulence and pathogenic. Identification of the genetic variation of *B. bassiana* strains infecting the different hosts would improve our understanding of the differences towards the hosts, and may help clarify the potential virulence of *B. bassiana* against the insects. Recently, several researches have been performed to investigate the genetic variation within the pathogen originated from different hosts using re-sequencing technology and bioinformatics [13–15]. Based on that, it is of great significance to investigate the genomic alterations of 17 *B. bassiana* isolates of the fungus with different levels of virulence to other Fungi in the present study.

Firstly, 17 strains of *B. bassiana* originated from different hosts were sequenced by genome re-sequencing. In general, the alignment rate obtained by comparing the assembled sequences is not comparable, and the mutation frequency cannot be known. For example, if a mutation occurs at a partial site of a sample, i.e. a heterozygote appears, the mutation cannot be counted. Therefore, we chose to match the quality-controlled clean reads to the reference genome of *B. bassiana* strain ARSEF 2860 [16], which identify the difference loci and calculate their genotype frequency, so as to be more accurate. The results showed approximately 71.22% of the cleaned reads were mapped to the *B. bassiana* strain ARSEF 2860, and a total of 2,779,949 SNPs, 884,811 Indels, and 4163 SVs, were identified in seventeen genomes when compared to the reference genome. As the most credible genome-wide genetic markers, single-nucleotide polymorphisms (SNPs) have been widely applied to characterize potentially adaptive genetic variation [17]. Herein, the proportion of nonsynonymous SNPs among all the SNPs is 29.51%, which is supported by Valero-Jiménez’s work of 87,119 SNPs between Bb5078 and Bb8028, and 467,292 SNPs between Bb4305 and Bb8028 [18], revealing a high level of genetic diversity among different strains of *B. bassiana*. Among the Indels, the number of InDel-Ins variants (423,409, 47.85%) is almost equal to the InDel-Del variants (461,402, 52.15%), and most of the variants were located in the upstream region of the genes (79.15%). Furthermore, structural variation is known to be a key component of phenotypic diversity in organisms [19]. In this study, all types of the identified SVs were inter-chromosomal translocations (CTXs), which may be due to the incomplete splicing of genomes caused by the existence of a considerable amount of gaps in the fungus genome [20].

Secondly, a hierarchical clustering analysis was carried out on the top 50 genes with missense SNPs in 17 samples. The results showed that there was no significant difference in the frequency of missense SNP mutations among different hosts, which revealing the population identity of the seventeen sequenced *B. bassiana* isolates. However, significant nucleotide variations were identified in four genes (gene_8222, gene_358, gene_6727, and gene_5020) in all samples, and two of them encoding the common protein non-ribosomal peptide synthase (NRPS, gene_8222 and gene_6727). NRPS is a key mechanism for the biosynthesis of bioactive metabolites in fungi and plays an important role in the production of fungaltoxin. For example, NRP synthetase Pes3 has been found to contribute to *Aspergillus. fumigatus* virulence, and disruption of *pes3* resulted in an augmented virulence phenotype in invertebrate for invasive aspergillosis. NRP synthetase mgoA has a role in the virulence of *Pseudomonas. syringae* pv. *Syringae*, and A Tn5 mutant disrupted in mgoA was defective in mangotoxin production and virulence. NRP synthetase gliP was predicted to be involved in gliotoxin production, and mutants with a disrupted gliP locus failed to produce gliotoxin [21–23]. Based on these findings, nonribosomal peptide synthesis (NRPS) is considered to be a documented virulence factor for fungi, its mutations affect the different virulence of the hosts. Importantly, the two genes encoding NRPS in this study showed more missense mutations in 17 *B. bassiana* trains, which may be closely related to the maintenance of the pathogenesis during the evolution process, but the specific virulence effects of each mutation need to be further explored. Furthermore, GO annotations showed that the functions of mutated genes were mainly related to binding, catalytic activity, extracellular region, metabolic process, and nucleic acid binding transcription transporter activity. Mutated genes involved in binding, transport and other biological processes may be associated with increased interaction between virulence associated proteins and hosts when *B. bassiana* exerts a toxic infection. Additionally, among these groups, the variation involved in the metabolic process may be related to the production of antibacterial compounds, it is very important for *B. bassiana* to resist microbial competition in the process of killing insects [24].

Thirdly, the phylogenetic relationships and principal component analysis (PCA) between isolates of *B. bassiana* were determined using the whole missense SNPs. Three distinct *B. bassiana* clusters were formed, namely the isolates of S2\S3\S4\S5\S6\S8\S14\S19, the isolates of S2\S9\S10\S13\S15\S18\S19\S20\S21, and the isolates of S7/S11﻿. Previously, the study of Ghikas et al. revealed that seven phylogenetic clusters in *B. bassiana* that consisted of isolates which had common climate features but did not exhibit any geographic distribution [25]. Our finding is partly in agree with the earlier suggestions that *B. bassiana* is not monophyletic, but rather is composed of several clusters that are morphologically identical [25–27], which jointly confirmed a high genetic diversity of *B. bassiana*. Additionally, the results showed that similar mutations occurred in *B. bassiana* strains in each cluster during evolution, which indicated a close relationship among *B. bassiana* isolates isolated different hosts in each cluster, but the relationship between the virulence of *B. bassiana* isolates in each cluster is not clear and further studies are needed. Furthermore, in the PCA plot, an obvious separation of three clusters was found, which also revealed a clear genetic differentiation among these 17 samples.

In previous study, genes with a signature of positive selection is possible to result in the adaptation to a dynamic environment and may be associated with fungi virulence [18]. Herein, we calculated the Ka/Ks values to identify the genes subjected to selection in the evolution of *B. bassiana*. Finally, a total of 7050 genes with Ka/Ks > 1 and 720 genes with Ka/Ks < 1 are confirmed, respectively. Among genes predicted to be under strong purification selection, there is enrichment for GO categories involving NAD + ADP-ribosyltransferase activity, chromatin, chromatin assembly or disassembly, chitin catabolic process, and actin cytoskeleton. In *Salmonella*, *spv* genes can establish successful systemic infection in experimental animals, and SpvB virulence-associated protein has recently been shown to contain the ADP-ribosyltransferase domain, suggesting that NAD + ADP-ribosyltransferase activity is associated with the regulation of virulence [28]. Chromatin is densely packed with the nucleosome that contains the genetic information in eukaryotes. Many events involve chromatin alterations that are crucial for virulence and have been considered to be a hopeful antifungal targets. For example, chromatin function affects pathogenicity of the most prevalent human fungal pathogen *Candida albicans* [29]. Earlier study has confirmed that the important role of drastic actin cytoskeleton reorganization in various different aspects of host-pathogen interaction [30]. Chitin is one of the main components of insect cuticle that acts as the first barrier against fungal pathogens. The entomopathogenic fungi *B. bassiana* could secrete several chitin-degrading enzymes, such as Endo chitosanase and Chitinase D [31], which are the critical cuticle-degrading enzymes and act synergistically with proteases to digest insect cuticle, and thus cause the enhancement of *B. bassiana* virulence [32]. Additionally, KEGG pathways analysis suggested the positively selected genes were highly enriched in glycosphingolipid biosynthesis-ganglio series, methane metabolism, proximal tubule bicarbonate reclamation, two component systems, and carbon fixation in photosynthetic organisms with some important physiological functions. Of which, two component systems (TCSs) are widely distributed in various bacteria and play important roles in the organisms in the face of different external stimuli via controlling gene expression [33]. In the present study, seven genes among these 17 *B. bassiana* strains with positive selection were abundantly enriched in this pathway. Increasing studies have revealed the involvement of this pathway in regulating resistance processes and diverse virulence in a wide range of pathogenic bacteria, such as *Mycobacterium tuberculosis* [33], *Salmonella typhimurium* [34], *Staphylococcus aureus* [35], *Pseudomonas aeruginosa* [36]. Taken together, GO and KEGG annotation analysis identified some positive selection genes associated with these above functional processes in the present study, which may serve as potential factors for virulence mechanisms, providing new support for studying of the virulence of *B. bassiana* strains*.*

Finally, a comprehensive protein domain analysis was performed on 720 positively selected genes with missense mutations. Significant differences in 13 domains encoded by 13 protein-coding genes were identified. Among these, three domain proteins involved in the host-pathogen interaction, such as ankyrin repeat domain protein, Metallophos domain protein and MutS domain V that were encoded by gene-7731, gene-5369, and gene-10,314 may affect host-pathogen interactions. For example, Zahrt et al. found that the alteration in virulence on *Salmonella* was the direct result of the inactivation of both of the *mutS* and *recD* gene products, however, *S. typhimurium* carrying mutations in either one of these genes behaved similarly to the wild-type strain and without attenuating virulence [37]. Ankyrin repeats are essential to regulate protein-protein interactions involved in a variety of host processes, including cytoskeletal motility, tumor suppression, and transcriptional regulation [38, 39]. Besides, Lamb et al. reported that myxoma virus as an effective vertebrate pest control agent, ANK-R mutant can both activate NF-κB pathway and engender virus-induced apoptosis [40]. The Metallophos domain is located in the conserved region of the Ser/Thr protein phosphatase gene, which is the central mediator of phosphorylation-dependent signals in various pathogenic bacteria and eukaryotes and is responsible for achieving metabolic control [41]. Earlier research has unveiled the important role of Ser/Thr phosphorylation in the virulence of *Mycobacterium tuberculosis*, which could regulate many intracellular metabolic processes and interfere with signaling pathways of the infected host cell [42]. In summary, based on the correlation between the virulence and these domains from different hosts, the altered protein domain in these 17 *B. bassiana* strains may be closely related to its plasticity of virulence. Besides, other genes deletion or genes acquisition may also be critical in virulence evolution. Of course, further study is needed in the future.

## Conclusion

According to the plasticity and variability of fungal virulence, we sequenced and compared the genome of seventeen *B. bassiana* isolates obtained from different insect hosts. Subsequently, the relationship between stable genetic variation and virulence function shared by 17 strains of *B. bassiana* in different hosts, and the correlation between mutated genes and virulence effect of *B. bassiana* were analyzed. A number of genes with stable mutations in these *B. bassiana* isolates have been established. Among them, some key factors including NRPS mutations, domain changes of ankyrin repeat domain protein, Metallophos domain protein, and MutS domain occurred in *B. bassiana* isolates may be crucial for the virulence of *B. bassiana*. Besides, the 720 positive selection genes and the top 50 genes with missense mutations were significantly enriched in some important biological processes, and pathways may also be related to the virulence of *B. bassiana* against hosts. The study provided the relevant theoretical basis for targeted changes in the virulence effect of *B. bassiana.* In this case, we think the virulence of *B. bassiana* might be influenced by natural variation in the process of evolution, which may benefit future molecular investigations of insect-fungus interactions and promote the development of *B. bassiana* as cost-effective and sustainable mycoinsecticides. However, do these genetic mutations really play a role in virulence or how do it regulate virulence in biological processes, which is interested and will need to further study.

## Methods

### Fungal strains and culture conditions

This study employed seventeen isolates of *B. bassiana* that were obtained from 17 different insects (Table S4). All isolates were preserved on Sabouraud dextrose agar (SDAY: 4% glucose, 1% peptone, 1.5% agar and 1% yeast extract) and maintained at 25 °C.

### Genome sequencing and mapping

Genomic DNA of 17 isolates of *B. bassiana* was extracted using the CTAB method as the previous report [43]. DNA concentration and purity were checked on NanoDrop (Thermo Fisher Scientific Inc. Waltham, MA, USA), and the qualified DNAs were used for library construction. Sequencing libraries for each individual were established using the standard Illumina protocol (Illumina, San Diego, CA, USA) and sequenced using HiSeq 2000 instruments (Illumina inc., San Diego, CA, USA) according to the manufacturer’s protocol by Sangon Biotech (Shanghai) Co., Ltd., Shanghai, China. The generated raw reads were processed using the FASTX-Toolkit version 0.0.13 (http://hannonlab.cshl.edu/fastx_toolkit) to filter the low-quality reads (< 10), reads shorter than 50 nt and duplicated reads [44]. After the filtering, the high quality reads were extracted and mapped to the reference genome of *B. bassiana* strain ARSEF 2860 [16]. The alignment processing was conducted using BWA (version V0.7.12-r1039) with default parameters [45].

### SNP and Indel detection

Picard tools version 1.92 (http://picard.sourceforge.net) was used to discard duplicates, and the obtained BAM files were further processed using SAMtools 1.4.1 (samtools.sourceforge.net) [46]. SNPs and indel calling was performed with the GATK v2.4–9 (software broadinstitute.org/gatk/) UnifiedGenotyper [47]. After that, an initial GATK VariantFiltration step was performed with the filtering conditions: For SNPs: with the filter expression ‘QD < 2.0 || FS > 60.0 || MQ < 40.0 || HaplotypeScore > 13.0 || MappingQualityRankSum < -12.5 || ReadPosRankSum < -8.0’; for Indel: QD < 2.0 || FS > 200.0 || ReadPosRankSum < − 20.0. The annotations for genes with SNPs and Indels were completed using the software SNPEFF (version 4.3 k) variant effect prediction (http://snpeff.sourceforge.net/) [48].

### Species clustering analysis

To determine the evolutionary relationship among *B. bassiana* originates from different hosts. The optimal nucleic acid replacement mode was found by modelfinder. Then a phylogenetic tree was conducted using iqtree with the bootstrapping of 100 replicates [49], and further presented using an R package, ggtree. PCA was carried out using the software Plink1.9 [50] on the missense SNPs that for all genotyped individuals, and presented using ggplot2.

### Ka/Ks analysis

The positively selected genes among the genomes of 17 *B. bassiana* isolates were searched using the bidirectional best hit (BBH) method [51]. The ratio of non-synonymous SNP (Ka) to synonymous SNP (Ks) in each gene was defined, including neutral (Ka/Ks = 1), negative (Ka/Ks > 1) and positive (Ka/Ks < 1) selection. The Ka and Ks in each missense mutant genes were calculated, and the ratio of Ka to Ks was estimated by the Codeml model of the program of phylogenetic analysis by maximum likelihood (PAML). To confirm functionally coherent gene-sets that showed Ka/Ks values significantly < 1, Gene Ontology (GO) enrichment analysis and Kyoto Encyclopedia of Genes and Genomes (KEGG) analysis were performed.

### Functional annotation

A hierarchical clustering analysis of the top 50 genes with missense SNPs was conducted by the clustergram function in the Matlab Bioinformatics toolbox with minor changes. GO analysis was performed with Fisher’s Exact Test and classified under three categories: biological process (BP), cellular component (CC), and molecular function (MF) [52]. Meanwhile, the detected positively selected genes were mapped to the terms of KEGG (http://www.genome.jp/kegg/) database to explore main biological functions [53], and the assignment of differential genes to different gene families was realized using Pfam (protein families) (http://pfam.xfam.org/) annotation [54].

### Structure variations (SVs) detection

Structural variants was determined using BreakDancer (v1.1.2) software [55], containing large fragments of SV types, including deletions (DEL), insertions (INS), inversions (INV), inter-chromosomal translocations (CTX), and so on.

### Protein domains analysis

To explore the effect of nonsynonymous SNPs in the positively selected genes on protein domains, we first identify the mutant amino acid sequences using Bioperl. Then, the hidden Markov model (HMM) profile for the NBS domain was applied to search the protein sequences in the *B. bassiana* database using hmmsearch in HMMER (v3.1b) with e-value < 0.00001 [56].

## Supplementary information

**Additional file 1: Table S1.** Quality control results of sequencing data of 17 *B. bassiana* strains. **Table S2.** Mutation information of the seven positively selected genes that are enriched in the two component systems. **Table S3.** The formation of the structure variants shared by seventeen B. bassiana isolates genomic sequences. **Table S4.** The information of the hosts of seventeen B. bassiana isolates. **Table S5.** The number of genes shared between the 17 isolates presented in each category in Fig. 1.

## Data Availability

The datasets generated and analysed during the current study are available in the SRA database of National Center for Biotechnology Information [https://www.ncbi.nlm.nih.gov/sra/PRJNA626696 and SRA accession: PRJNA626696].

## Abbreviations

B. bassiana: Beauveria bassiana
PCA: Principal component analysis
SNPs: Single-nucleotide polymorphisms
CTXs: Inter-chromosomal translocations
NRPS: Nonribosomal peptide synthesis
TCSs: Two component systems
BBH: Bidirectional best hit
GO: Gene Ontology
KEGG: Kyoto Encyclopedia of Genes and Genomes
BP: Biological process
CC: Cellular component
MF: Molecular function
SVs: Structure variations
DEL: Deletions
INS: Insertions
INV: Inversions
HMM: Hidden Markov model
