# Supplementary material for: Influence of genetic diversity of seventeen Beauveria bassiana isolates from different hosts on virulence by comparative genomics
Source: BMC Genomics. 2020 Jun 30;21:451. doi: 10.1186/s12864-020-06791-9 (PMC7329388; doi:10.1186/s12864-020-06791-9)
Supplement: Supplementary file 1 — Additional file 1: Table S1. Quality control results of sequencing data of 17 B. bassiana strains. Table S2. Mutation information of the seven positively selected genes that are enriched in the two component systems. Table S3. The formation of the structure variants shared by seventeen B. bassiana isolates genomic sequences. Table S4. The information of the hosts of seventeen B. bassiana isolates. Table S5. The number of genes shared between the 17 isolates presented in each category in Fig. 1. [file 12864_2020_6791_MOESM1_ESM.docx]

**Table S1 Quality control results of sequencing data** **of 17 *B. bassiana* strains**

| Sample | Raw Reads | Raw Bases | Raw Q20% | Clean Reads | Clean Bases | Clean rate |
| --- | --- | --- | --- | --- | --- | --- |
| S2_R1 | 3614771 | 542215650 | 99.85 | 2893673 | 402220547 | 0.800513504 |
| S2_R2 | 3614771 | 542215650 | 97.70 | 2893673 | 399326874 | 0.800513504 |
| S3_R1 | 2965561 | 444834150 | 99.87 | 2376759 | 327992742 | 0.801453418 |
| S3_R2 | 2965561 | 444834150 | 97.66 | 2376759 | 320862465 | 0.801453418 |
| S4_R1 | 4434296 | 665144400 | 99.85 | 3237968 | 456553488 | 0.730210162 |
| S4_R2 | 4434296 | 665144400 | 96.41 | 3237968 | 424173808 | 0.730210162 |
| S5_R1 | 4943534 | 741530100 | 99.88 | 4098149 | 557348264 | 0.82899177 |
| S5_R2 | 4943534 | 741530100 | 98.05 | 4098149 | 561446413 | 0.82899177 |
| S6_R1 | 5167071 | 775060650 | 99.88 | 4281376 | 582267136 | 0.828588576 |
| S6_R2 | 5167071 | 775060650 | 97.70 | 4281376 | 590829888 | 0.828588576 |
| S7_R1 | 7388636 | 1108295400 | 99.89 | 6316839 | 865406943 | 0.854939802 |
| S7_R2 | 7388636 | 1108295400 | 97.99 | 6316839 | 865406943 | 0.854939802 |
| S8_R1 | 11254124 | 1688118600 | 99.93 | 9916773 | 1338764355 | 0.881167917 |
| S8_R2 | 11254124 | 1688118600 | 98.26 | 9916773 | 1348681128 | 0.881167917 |
| S9_R1 | 15042841 | 2256426150 | 99.93 | 12774742 | 1788463880 | 0.849224026 |
| S9_R2 | 15042841 | 2256426150 | 98.11 | 12774742 | 1737364912 | 0.849224026 |
| S10_R1 | 9190258 | 1378538700 | 99.87 | 7679800 | 1067492200 | 0.835645746 |
| S10_R2 | 9190258 | 1378538700 | 97.68 | 7679800 | 1036773000 | 0.835645746 |
| S11_R1 | 10635026 | 1595253900 | 99.89 | 8640590 | 1175120240 | 0.812465339 |
| S11_R2 | 10635026 | 1595253900 | 97.86 | 8640590 | 1175120240 | 0.812465339 |
| S13_R1 | 9005595 | 1350839250 | 99.92 | 7644899 | 1062640961 | 0.848905486 |
| S13_R2 | 9005595 | 1350839250 | 97.73 | 7644899 | 1047351163 | 0.848905486 |
| S14_R1 | 12020859 | 1803128850 | 99.94 | 10485622 | 1436530214 | 0.872285583 |
| S14_R2 | 12020859 | 1803128850 | 98.10 | 10485622 | 1447015836 | 0.872285583 |
| S15_R1 | 20018648 | 3002797200 | 99.93 | 17252744 | 2363625928 | 0.861833626 |
| S15_R2 | 20018648 | 3002797200 | 97.85 | 17252744 | 2363625928 | 0.861833626 |
| S18_R1 | 17933589 | 2690038350 | 99.92 | 15022660 | 2088149740 | 0.837682853 |
| S18_R2 | 17933589 | 2690038350 | 97.34 | 15022660 | 2058104420 | 0.837682853 |
| S19_R1 | 36557855 | 5483678250 | 99.95 | 31532295 | 4004601465 | 0.862531322 |
| S19_R2 | 36557855 | 5483678250 | 97.63 | 31532295 | 4193795235 | 0.862531322 |
| S20_R1 | 34133484 | 5120022600 | 99.95 | 30424817 | 4137775112 | 0.891348126 |
| S20_R2 | 34133484 | 5120022600 | 98.26 | 30424817 | 4137775112 | 0.891348126 |
| S21_R1 | 25603570 | 3840535500 | 99.94 | 22268579 | 2939452428 | 0.869745079 |
| S21_R2 | 25603570 | 3840535500 | 97.86 | 22268579 | 3028526744 | 0.869745079 |

**Table S2 Mutation information of the seven positively selected genes that are enriched in the two component systems**

| Gene | Chr | Pos | Ref | Change | Sample. Number |
| --- | --- | --- | --- | --- | --- |
| gene_664 | scaffold_00001 | 2032618 | T | C | 13 |
|  | scaffold_00001 | 2032766 | G | C | 16 |
|  | scaffold_00001 | 2033056 | T | C | 16 |
|  | scaffold_00001 | 2032981 | A | G | 17 |
|  | scaffold_00001 | 2033035 | G | C | 17 |
|  | scaffold_00001 | 2033094 | A | T | 17 |
|  | scaffold_00001 | 2033095 | C | T | 17 |
| gene_2709 | scaffold_00005 | 1371959 | A | C | 17 |
| gene_3056 | scaffold_00006 | 954321 | G | A | 2 |
|  | scaffold_00006 | 954313 | A | T | 3 |
|  | scaffold_00006 | 954315 | A | G | 3 |
|  | scaffold_00006 | 954288 | G | C | 11 |
|  | scaffold_00006 | 954339 | A | T | 11 |
|  | scaffold_00006 | 954251 | C | G | 16 |
|  | scaffold_00006 | 954121 | G | A | 17 |
|  | scaffold_00006 | 954225 | T | G | 17 |
| gene_3362 | scaffold_00007 | 432919 | C | T | 15 |
|  | scaffold_00007 | 432865 | A | C | 17 |
| gene_7254 | scaffold_00023 | 457092 | G | A | 17 |
| gene_7433 | scaffold_00025 | 28843 | A | T | 16 |
|  | scaffold_00025 | 28972 | G | T | 17 |
| gene_8336 | scaffold_00034 | 13235 | G | A | 1 |
|  | scaffold_00034 | 12720 | C | T | 17 |
|  | scaffold_00034 | 13406 | A | G | 17 |

**Table S3 The formation of the structure variants shared by seventeen *B. bassiana* isolates genomic sequences**

| Chromosome_1 | Pos_1 | Chromosome_2 | Pos_2 | Sample_No. | | Samples |
| --- | --- | --- | --- | --- | --- | --- |
| scaffold_00054 | 10022 | scaffold_00060 | 72882 | 3 | S15;S21;S5 | |
| scaffold_00085 | 33205 | scaffold_00093 | 5697 | 3 | S13;S21;S8 | |
| scaffold_00087 | 39245 | scaffold_00108 | 13942 | 3 | S14;S21;S5 | |
| scaffold_00093 | 11725 | scaffold_00097 | 9852 | 3 | S11;S21;S6 | |
| scaffold_00045 | 4174 | scaffold_00062 | 50722 | 4 | S15;S19;S20;S21 | |
| scaffold_00093 | 11449 | scaffold_00097 | 9852 | 4 | S10;S11;S7;S9 | |

**Table S4 The information of the hosts of seventeen *B. bassiana* isolates**

| Serial number | Host | Latin name |
| --- | --- | --- |
| S2 | Grub | Holotrichia diomphalia Bates |
| S3 | Soybean pod borer | *Leguminivora glycinivorella* Matsumura |
| S4 | Rice-stem borer | Chilo suppressalis Walker |
| S5 | European corn borer (French) | Ostrinia nubilalis Hubner |
| S6 | Oriental fruit moth | Grapholitha funebrana Treitscheke |
| S7 | Pine moth | *Dendrolimus punctatus* Walker |
| S8 | Chinese caterpillar fungus bat worm | *Hepialus armoricanus* |
| S9 | Asian particolored bat moth | *Phassus excrescens* Butler |
| S10 | European corn borer (Sinkiang) | Ostrinia nubilalis Hubner |
| S11 | Asiatic corn borer D1-5 | *Ostrinia furnacalis* Guenee |
| S13 | Phytomyza nigra Meigen, 1830 | Chromatomyia nigra Meigen |
| S14 | Leafhopper | Nephotettix bipunctatus Fabricius |
| S15 | Stink bug | Aspongopus chinensis Dallas |
| S18 | Conogethes punctiferalis | *Dichocrocis chlorophanta* Butler |
| S19 | Weevil | *Cyrtotrachelus longimanus* |
| S20 | Rice-stem borer | Ostrinia nubilalis Hubner |
| S21 | Patato beetle | *Leptinotarsa decemlineata* Say |

**Table S5** **The number of genes shared between the 17 isolates presented in each category in figure 1**

| Type | S2 | S3 | S4 | S5 | S6 | S7 | S8 | S9 | S10 | S11 | S13 | S14 | S15 | S18 | S19 | S20 | S21 |
| --- | --- | --- | --- | --- | --- | --- | --- | --- | --- | --- | --- | --- | --- | --- | --- | --- | --- |
| upstream_gene_variant | 130701 | 127903 | 131489 | 134669 | 132817 | 133399 | 130586 | 125946 | 126455 | 131829 | 124390 | 127733 | 126760 | 120617 | 115052 | 122435 | 123018 |
| synonymous_variant | 115467 | 114535 | 116857 | 116602 | 116319 | 116665 | 116818 | 115345 | 115592 | 117076 | 114865 | 116203 | 115212 | 113821 | 107062 | 110678 | 113443 |
| missense_variant | 49302 | 48784 | 49518 | 49440 | 49169 | 49094 | 48930 | 47993 | 48000 | 48938 | 47577 | 48408 | 47805 | 46923 | 44423 | 45993 | 47066 |
| downstream_gene_variant | 12313 | 11895 | 11938 | 12615 | 12307 | 12524 | 12297 | 11725 | 11864 | 12397 | 11703 | 11966 | 11891 | 11299 | 10704 | 11302 | 11461 |
| splice_region_variant&intron_variant | 3189 | 3168 | 3140 | 3189 | 3204 | 3148 | 3078 | 3039 | 3073 | 3138 | 3052 | 3084 | 3070 | 3007 | 2829 | 2954 | 3013 |
| intergenic_region | 2146 | 2070 | 2130 | 2247 | 2257 | 2268 | 2232 | 2155 | 2122 | 2246 | 2094 | 2159 | 2147 | 2029 | 1893 | 2016 | 2123 |
| splice_region_variant&synonymous_variant | 463 | 430 | 434 | 437 | 420 | 433 | 414 | 410 | 422 | 432 | 413 | 417 | 416 | 408 | 390 | 411 | 406 |
| intron_variant | 462 | 443 | 464 | 458 | 495 | 509 | 504 | 503 | 474 | 497 | 471 | 469 | 509 | 478 | 455 | 487 | 462 |
| stop_gained | 290 | 305 | 306 | 276 | 282 | 282 | 273 | 271 | 276 | 268 | 271 | 264 | 270 | 262 | 240 | 256 | 257 |
| start_lost | 31 | 30 | 27 | 30 | 24 | 22 | 29 | 22 | 24 | 23 | 23 | 25 | 24 | 23 | 22 | 22 | 23 |
| missense_variant&splice_region_variant | 185 | 182 | 182 | 176 | 179 | 179 | 181 | 160 | 168 | 166 | 165 | 166 | 167 | 156 | 149 | 152 | 157 |
| splice_region_variant&stop_retained_variant | 155 | 148 | 162 | 147 | 159 | 153 | 149 | 147 | 143 | 151 | 147 | 153 | 143 | 144 | 136 | 141 | 144 |
| splice_donor_variant&intron_variant | 66 | 67 | 61 | 73 | 73 | 68 | 67 | 64 | 65 | 69 | 68 | 67 | 68 | 64 | 64 | 65 | 66 |
| stop_lost&splice_region_variant | 61 | 67 | 63 | 64 | 65 | 63 | 59 | 60 | 58 | 61 | 59 | 57 | 59 | 55 | 49 | 55 | 55 |
| splice_acceptor_variant&intron_variant | 57 | 47 | 49 | 45 | 50 | 46 | 49 | 40 | 41 | 51 | 42 | 49 | 48 | 41 | 42 | 45 | 45 |
| stop_gained&splice_region_variant | 7 | 4 | 7 | 6 | 8 | 5 | 6 | 5 | 6 | 5 | 6 | 6 | 5 | 5 | 5 | 5 | 4 |
| initiator_codon_variant | 6 | 5 | 8 | 5 | 5 | 4 | 5 | 4 | 5 | 4 | 6 | 5 | 5 | 5 | 4 | 4 | 4 |
